# Bipolar Disorder VI: Long-Term Follow-Up of Suicide Attempts and Prophylactic Effect of Lithium Treatment in Bipolar Affective Disorder

**DOI:** 10.1016/j.bpsgos.2026.100735

**Published:** 2026-04-21

**Authors:** Per-Olof Nylander, Erik Lexne, Christer Lehman, Lars Brudin, Finn Bengtsson

**Affiliations:** a Linköping University, Linköping, Sweden; b Academic Clinic of Färjestaden, Färjestaden, Sweden; c Forensic Psychiatric Regional Clinic, Sundsvall, Sweden; d Department of Medical and Health Science, Linköping University, Linköping, Sweden; e Division of Clinical Chemistry and Pharmacology, Department of Biomedical and Clinical Sciences, Linköping University, Linköping, Sweden

**Keywords:** Bipolar disorder, Episodes, Family history, Lithium treatment, Long-term follow-up, Suicide attempts

## Abstract

**Background:**

Bipolar disorder (BD) is a common illness that is highly associated with suicide attempts (SAs) and suicide. There have been few long-term follow-up studies exploring characteristics of SAs among patients with bipolar disorder (BPs).

**Methods:**

BPs (*n* = 192) diagnosed using DSM-IV criteria were recruited and followed up for approximately 25 years, comparing patients without and with SA on several relevant clinical variables. The effect of lithium treatment was also studied.

**Results:**

BPs with SAs were more commonly female (*p* = .047), had poorer treatment response to lithium (*p* = .018) and poorer compliance (*p* = .008), were younger (*p* = .010), and had earlier age of onset (*p* < .001). A family history of first- and/or second-degree relatives with affective disorders was more common in BPs with SAs (*p* = .028), who also more often had a first-degree relative with an affective disorder (*p* = .020). A family history of suicide was more common in BPs with SAs (*p* = .043). There were more total episodes of the disease (*p* < .001), episodes per year (*p* = .002), total depressive episodes (*p* = .001), and depressive episodes per year (*p* < .001) in BPs with SAs. While off lithium, there were more depressive episodes in BPs with SAs (*p* = .011). While on lithium, BPs with SAs had more episodes (*p* = .002), episodes per year (*p* = .001), depressive episodes (*p* = .001), depressive episodes per year (*p* = .004), hypomanic episodes (*p* = .034), and hypomanic episodes per year (*p* = .008). Multivariate logistic regression revealed significant risk factors in age of onset (*p* = .001) and total number of depressive episodes/year (*p* = .003).

**Conclusions:**

BPs with SAs have a more severe form of BD and less protective effect of lithium treatment.

## Plain Language Summary

A total of 192 bipolar patients (BPs) with or without suicide attempts (SAs) were followed up for approximately 25 years, with assessments of several clinical variables. The effect of lithium treatment was also studied. Multivariate logistic regression revealed significant risk factors in age of onset of BPs (*p* = .001) and total number of depressive episodes/year (*p* = .003). BPs with SAs had a more severe form of BD and less protective effect of lithium treatment.

Bipolar disorder (BD) is a widespread, common, chronic affective disorder with a significant impact on quality of life (1,2). Suicidal behavior including suicidal thoughts, suicide attempts (SAs), and death by suicide are common in BD (3, 4, 5, 6). Approximately 29% of patients with BD (BPs) attempt suicide at least once during their lifetime (7). In clinical samples, SA is estimated to occur in 25% to 56% of BPs (3,6), and death by suicide is estimated to occur in 10% to 19% of BPs (3,6). However, in more recent literature, the percentages of suicidal behavior in BPs appear to be somewhat lower (8).

Previous studies of BD have shown that both SAs and death by suicide occur most commonly within the first few years after age of onset (AOO) (5,9,10). A recent study found that approximately 20% of attempts occurred close to the AOO, while approximately 50% occurred within 5 years, 65% within 10 years, and 90% within 25 years (11).

There are several known risk factors for SAs in BPs including previous SAs (3,5), female gender (12, 13, 14, 15), presence of depressive episodes (8,10,15), severe depressive and dysphoric-agitated mixed phases, and repeated severe depressive episodes (5). One previous study also found that BPs with SAs had more than twice as many depressive episodes during their lifetimes compared with patients without SAs (16).

Several studies (17, 18, 19, 20) have suggested that early AOO is associated with a more severe form of BD associated with psychotic (or schizoaffective) features (21), a higher proportion of manic episodes (22,23), a poorer prognosis (17,24,25), and a poorer prophylactic response to lithium therapy (20,25,26). Research has also shown that early AOO in BD is associated with SAs (8,14,15,20, 21, 22, 23, 24, 25, 26, 27, 28, 29).

Previous studies have found that a family history (FH) of both suicide (30) and SAs are risk factors for the presence of SAs in BD (31). An FH of affective disorders (ADs) has also been found to be a risk factor for SAs in BD (10,13,30,32, 33, 34). Another study found that a familial psychiatric history in first-degree relatives was associated with several disease severity measures such as the need for hospitalizations, SAs, and earlier AOO (35).

Lithium is effective in preventing depressive and especially manic episodes (36, 37, 38, 39, 40, 41, 42) and may prevent SAs (43,44) in as many as 20% to 82% of BD cases (45,46). Studies have shown that lithium is more effective in patients with an FH of BD (47, 48, 49, 50, 51, 52). Mixed cohorts including a range of affective disorders with small samples and short follow-up time have found variable associations of lithium’s prophylactic effect among patients with an FH of any AD (52, 53, 54). Other studies with a more homogenous BD population have found poorer lithium prophylactic outcome among BPs with an FH of AD (19,20,55,56), and a recent meta-analysis of clinical risk factors confirmed that only FH of BD was associated with lithium response (57).

The aim of this study was to investigate features of and risk for SAs and relevant differences between patients with and without SAs during the course of BD. For this purpose, we recruited 192 BPs from lithium dispensaries in northern Sweden in a long-term follow-up study of approximately 25 years.

## Methods and Materials

Altogether, 219 BPs from 3 lithium dispensaries in northern Sweden (Umeå, Sundsvall, Härnösand) were recruited for this study. Patients with a DSM-IV (58) diagnosis of alcoholism or drug abuse, epilepsy, or intellectual disability were excluded. There were no other comorbid diagnoses such as anxiety disorders, neuropsychiatric disorders, or personality disorders.

For all included patients, medical records were available containing previous psychiatric illness; SAs; episodes of BD, AOO, FH; and previous and ongoing medical treatment. Patients were continuously followed from date of onset, and all episodes of the disease have been documented in case record forms at the lithium dispensaries by experienced psychiatrist colleagues. All previously documented data, including diagnoses, were then retrospectively reviewed and confirmed by two experienced psychiatrists involved in this investigation (P-ON and CL). All patients were interviewed by using the Present State Examination (59), and all data were checked by the Operational Criteria Checklist (60). All interviews were conducted simultaneously when all previously documented data were collected for the study. Interrater reliability was 1.0 for diagnoses, episodes, AOO, and all other clinical variables. The very high interrater reliability was most likely the result of long-term collaboration between P-ON and CL, who had developed a similar basis for making clinical judgments. The interrater reliability was performed by P-ON and CL, who first made a separate judgment of all data for a particular patient. Then these findings were compared for each patient, and outcomes were found to be identical in all cases. In any case, the proven good consensus between the two raters is considered a major strength of the current investigation.

Bipolar AD type I and II were diagnosed using DSM-IV criteria (58). An affective episode was defined according to DSM-IV and Research Diagnostic Criteria (RDC) (61) for major depressive, manic, or hypomanic episodes, respectively, requiring either hospitalization or treatment as an outpatient. AOO was defined by the first episode of depression, hypomania, or mania. We also obtained the number of years from AOO until fulfilling criteria for a diagnosis of BD.

Patients were divided into 2 groups, those with and those without a history of SA. It could be argued that some BPs may harm themselves or even die by accidental trauma that is not related to an SA. However, we only included BPs who had an intended SA, and our figure of an overall rate of this by 26.6% is in good agreement with previous studies (3, 4, 5, 6). In addition, no recruited patients had a comorbid personality disorder, which is important because self-harm behavior is not uncommon in this group of individuals.

FH of AD (unipolar or bipolar) was obtained from medical records, by semistructured interviews of patients and relatives (by P-ON and CL), retrieving also FH-RDC (62). Only patients with an FH of AD among first- and/or second-degree relatives were defined as having an FH of AD.

Information was collected regarding the total number of episodes and frequency of depression, mania, and hypomania from AOO until the end of the study. The patient journey was divided into 1) the time before initiation of lithium therapy, 2) the time on lithium after initiation, and 3) the time off lithium after initiation. In this way, it was possible to measure episode frequency during each period. Percentage lithium response was calculated by comparing episodes per year while off or on lithium treatment, respectively. Compliance was defined by measure of serum lithium levels and medical records. Poor compliance was defined if chart notes indicated discontinuation of medication or if serum levels (S-levels) of lithium (S-lithium) were found to be low or to fluctuate over time. The latter is defined as if S-lithium displayed a significant variation despite an unchanged dose of lithium or if a change in lithium dose was not followed by an expected change in the concentration of S-lithium without an alternative explanation (e.g., interactions with drugs, extensive diarrhea). S-lithium was measured for all patients on lithium treatment every third month in parallel with a clinical judgment. This was the standard monitoring protocol used at the lithium dispensaries where all BPs were treated. According to the guidelines used at the lithium dispensaries, the therapeutic interval for S-lithium for most patients was between 0.5 and 0.9 mmol/L, referring to trough levels (i.e., sample drawn before next dose) at steady state (usually achieved after 1 week on the same daily intake of the drug dose). The treatment goal was to have the optimal effect on as low a dose as possible. S-lithium above 1.2 mmol/L increases the risk for intoxication, and above 3.0 mmol/L levels are considered life-threatening and potentially lethal. For further reading regarding S-lithium levels, see (63).

Before initiation of lithium treatment, a depressive episode was in all cases treated with antidepressant drugs and, if needed, in combination with hypnotics and anxiolytics. Similarly, patients with a first episode of hypomania or mania were treated with antipsychotics. All medication was registered in the patients’ record form at the psychiatric clinic. When lithium treatment began, the lithium drug was used alone, i.e., not in combination with any other psychotropic drug, except for hypnotics and/or anxiolytics when clinically necessary.

Statistics were calculated with SPSS version 23 (IBM Corp.). Chi-squared with Fisher’s exact test (2-tailed) was used for analysis of prevalence based on categorical variables. For comparisons of continuous variables, Student’s *t* tests (normal distribution) and Mann-Whitney tests (non-normal distribution) were used. Univariate logistic regression (ULR) was performed on demographic data, AOO, FH of suicide and AD, episodes of the disease, and years of disease, while off or on lithium treatment, respectively. Multivariate logistic regression (MLR) was performed on risk factors with *p* < .1 from the ULR. A level with a *p* < .1 in ULR is usually chosen because a factor on this level may be significant in MLR. The overall significance level was preset to *p* < .05.

The ethical committee in Umeå (Sweden) approved the study (Dnr 92-045; principal investigator P-ON).

## Results

Of the 192 patients primarily included in the study, 27 patients were subsequently excluded due to intellectual disability (*n* = 1), epilepsy (*n* = 1), alcohol abuse (*n* = 1), schizophrenia (*n* = 1), and incomplete medical records (*n* = 23). Among the included patients, only 6 were diagnosed as rapid cyclers but did not interfere with the overall results. Further insights into the clinical phenotype of the study population are shown by the fact that 134 BPs had an index episode of depression, 12 BPs had an index episode of hypomania, and 46 BPs had an index episode of mania (see Table 1).

**Table 1:** Characteristics of 192 BD (Type I and II) Patients With and Without SAs During a Long-Term Follow-Up of Approximately 25 Years

	SA		*p*
	No, *n* = 141	Yes, *n* = 51	
Gender, Female	76 (53.9%)	36 (70.6%)	.047^a^
Age, Years	58.77 ± 15.07	52.51 ± 13.82	.007^a^
AOO, Years	32.51 ± 10.92	26.25 ± 9.43	<.001^a^
Diagnosis			
Bipolar diagnosis, years	8.23 ± 10.91	6.57 ± 6.98	n.s.
BD I	112 (79.4%)	43 (84.3%)	n.s.
BD II	29 (20.6%)	8 (15.7%)	n.s.
First Episode Depression	94 (66.7%)	40 (78.4%)	n.s.
Years Before Lithium	11.45 ± 11.27	11.38 ± 10.65	n.s.
Treatment Response			
Complete	31 (22.0%)	6 (11.8%)	.018^a^
Partial	97 (68.8%)	33 (64.7%)	
None	13 (9.2%)	12 (23.5%)	
Compliance, Poor	57 (40.4%)	32 (62.7%)	.008^a^

Values are presented as mean ± SD or *n* (%). Bipolar diagnosis indicates the time to fulfill the criteria for BD.

AOO, age of onset; BD, bipolar disorder; n.s., not significant; SA, suicide attempt.

a Statistical significance level.

Gender, diagnosis, first episode depression, lithium treatment response, and compliance in BPs with and without SAs are shown in Table 1. BPs with SAs were significantly more commonly female (*p* = .047). Lithium treatment response while on lithium was significantly (*p* = .018) associated with lower effectiveness in BPs with SAs. Furthermore, BPs with SAs demonstrated a highly significant (*p* = .008) poorer compliance.

Table 1 also shows age, AOO, years before lithium treatment was initiated, and time to meet criteria for BD in BPs with and without SAs. BPs with SAs were significantly (*p* = .007) younger and had a clearly significantly (*p* < .001) earlier AOO (approximately 26 vs. 32 years) compared with BPs without SAs.

FH of AD (first- and second-degree relatives) and suicide in BPs with and without SAs are shown in Table 2. An FH of first- and/or second-degree relatives with AD was significantly (*p* = .028) more common in BPs with SAs. They also had a significantly (*p* = .020) higher frequency of first-degree relatives with AD. Furthermore, an FH of suicide was significantly (*p* = .043) more common in BPs with SAs.

**Table 2:** Family History of ADs in 192 BD (Type I and II) Patients With and Without SAs During a Long-Term Follow-Up of Approximately 25 Years

	SA		*p*
	No, *n* = 141	Yes, *n* = 51	
Family History AD			
No	46 (32.6%)	8 (15.7%)	–
Yes			
1st and 2nd AD	95 (67.4%)	43 (84.3%)	.028^a^
1st AD	75 (53.2%)	37 (72.5%)	.020^a^
1st and 2nd BD	56 (39.7%)	24 (47.1%)	n.s.
1st BD	46 (32.6%)	20 (39.2%)	n.s.
1st and 2nd UD	39 (27.7%)	19 (37.3%)	n.s.
1st UD	29 (20.6%)	17 (33.3%)	n.s.
Family History Suicide	25 (17.0)	16 (31.4)	.043^a^

Values are presented as *n* (%).

1st, first-degree relative; 2nd, second-degree relative; AD, affective disorder; BD, bipolar disorder; n.s., not significant; SA, suicide attempt; UD, unipolar disorder.

a Statistical significance level.

Years, episodes, and episodes per year in BPs with and without SAs are shown in Table 3. Fifty-one patients had at least 1 SA, and 141 patients had no SAs. BPs with SAs had a significantly higher frequency of total episodes of disease (*p* < .001) and total episodes per year (*p* = .002) compared with BPs without SAs. BPs with SAs also had a significantly higher frequency of total number of depressive episodes (*p* = .001) and total depressive episodes per year (*p* < .001).

**Table 3:** Years, Episodes, and Episodes per Year in 192 Bipolar Disorder (Type I and II) Patients With and Without SAs During a Long-Term Follow-Up of Approximately 25 Years

	SA		*p*
	No, *n* = 141	Yes, *n* = 51	
Total			
Years of Follow-Up Time	24.98 ± 12.69	25.47 ± 11.62	n.s.
Episodes	12.65 ± 7.49	17.31 ± 9.04	<.001^a^
Episodes/Year	0.60 ± 0.38	0.76 ± 0.36	.002^a^
Depressive Episodes	7.00 ± 5.68	10.47 ± 7.37	.001^a^
Depressive Episodes/Year	0.31 ± 0.25	0.45 ± 0.26	<.001^a^
Hypomanic Episodes	2.91 ± 4.02	3.29 ± 3.13	n.s.
Hypomanic Episodes/Year	0.13 ± 0.16	0.15 ± 0.16	n.s.
Manic Episodes	2.87 ± 2.81	3.53 ± 3.18	n.s.
Manic Episodes/Year	0.16 ± 0.21	0.17 ± 0.15	n.s.

Before Lithium			
Years of Follow-Up Time	12.21 ± 10.35	13.13 ± 10.81	n.s.
Episodes	8.79 ± 5.75	10.71 ± 6.80	n.s.
Episodes/Year	1.22 ± 0.91	1.18 ± 0.75	n.s.
Depressive Episodes	5.01 ± 4.06	6.90 ± 5.48	.011^a^
Depressive Episodes/Year	0.60 ± 0.48	0.67 ± 0.46	n.s.
Hypomanic Episodes	1.69 ± 2.73	1.55 ± 2.31	n.s.
Hypomanic Episodes/Year	0.22 ± 0.37	0.13 ± 0.16	n.s.
Manic Episodes	2.09 ± 2.15	2.27 ± 1.93	n.s.
Manic Episodes/Year	0.41 ± 0.59	0.38 ± 0.46	n.s.

On Lithium			
Years of Follow-Up Time	12.69 ± 8.24	12.35 ± 8.14	n.s.
Episodes	3.84 ± 4.26	6.63 ± 6.21	.002^a^
Episodes/Year	0.37 ± 0.39	0.61 ± 0.54	.001^a^
Depressive Episodes	1.96 ± 2.65	3.59 ± 4.07	.001^a^
Depressive Episodes/Year	0.19 ± 0.27	0.33 ± 0.36	.004^a^
Hypomanic Episodes	1.10 ± 1.88	1.80 ± 2.38	.034^a^
Hypomanic Episodes/Year	0.09 ± 0.15	0.16 ± 0.19	.008^a^
Manic Episodes	0.77 ± 1.32	1.24 ± 2.00	n.s.
Manic Episodes/Year	0.08 ± 0.17	0.12 ± 0.21	n.s.
S-Lithium Without Episode, mmol/L	0.61 ± 0.11	0.63 ± 0.10	n.s.
S-Lithium With Episode, mmol/L	0.60 ± 0.13	0.63 ± 0.12	n.s.

Values are presented as mean ± SD.

SA, suicide attempt; n.s., not significant; S-lithium, serum lithium.

a Statistical significance level.

While off lithium treatment (Table 3), there were no significant differences between the 2 patient groups except for a significantly higher frequency of depressive episodes (*p* = .011) in BPs with SAs compared with those without SAs. While on lithium therapy (Table 3), BPs with SAs had a significantly higher frequency of episodes of disease (*p* = .002), episodes per year (*p* = .001), depressive episodes (*p* = .001), depressive episodes per year (*p* = .004), hypomanic episodes (*p* = .034), and hypomanic episodes per year (*p* = .008) compared with those without SAs. There were no differences between the 2 groups in number of manic episodes, manic episodes per year, or measured S-lithium levels.

While on lithium treatment, episodes of disease and episodes per year were reduced by 56% and 70%, respectively, in BPs without SAs compared with 38% and 48%, respectively, in BPs with SAs (Table 3). Furthermore, depressive episodes and depressive episodes per year were reduced while on lithium therapy by 61% and 68%, respectively, in BPs without SAs compared with 48% and 51%, respectively, in BPs with SAs (Table 3). In our cohort of 192 BPs, 51 patients performed SA during a period of approximately 25 years with the disease. Of those patients, 39 (76.5%) performed an SA before lithium treatment was initiated and did not perform any further SAs while on lithium treatment. Twelve patients (24.5%) performed their first SA after initiation of lithium treatment.

ULR of risk factors for SAs in patients with BD are shown in Table 4. In general, significant risk factors identified were gender (*p* = .042), age (*p* = .014), AOO (*p* < .001), FH of suicide (*p* = .046), FH of AD (*p* = .026), total number of episodes of disease (*p* = .003), episodes per year (*p* = .010), and total number of depressive episodes per year (*p* = .002). Patients off lithium treatment showed no significant risk factors according to the ULR analysis. Further, patients on lithium treatment showed significant risk factors, including total number of episodes of disease (*p* = .002) and episodes per year (*p* = .002), in the URL analysis.

**Table 4:** ULR Analysis of Risk Factors for SAs in Bipolar Disorder

	*n*	SA, %	ULR	*p*
			OR (95% CI)	
Gender				
Female	112	32.1%	1.00	.042^a^
Male	65	23.1%	0.49 (0.24–0.97)	
Age Category, Years				
≤46	47	38.3%	1.00	.014^a^
47–58	46	26.1%	0.69 (0.51–0.93)	
59–68	48	29.2%	0.47 (0.26–0.86)	
>68	51	13.7%	0.33 (0.13–0.80)	
AOO, Years				
≤21	50	44.0%	1.00	<.001^a^
22–30	53	30.2%	0.53 (0.38–0.74)	
31–38	48	18.8%	0.28 (0.14–0.54)	
>38	41	9.8%	0.15 (0.05–0.40)	
Heredity for Suicide				
No	151	23.2%	1.00	.046^a^
Yes	41	39.0%	2.12 (1.01–4.43)	
Heredity for Affective Disorder				
No	54	14.8%	1.00	.026^a^
Yes	138	31.2%	2.60 (1.13–6.02)	
Total Number of Episodes				
≤9	60	13.3%	1.00	.003^a^
10–12	42	21.4%	1.56 (1.16–2.09)	
13–18	46	41.3%	2.42 (1.35–4.36)	
>18	44	34.1%	3.78 (1.56–9.11)	
Years of Disease				
≤15	49	24.5%	1.00	.996
16–24	48	31.3%	1.00 (0.75–1.33)	
25–34.25	47	23.4%	1.00 (0.56–1.78)	
>34.25	48	27.1%	1.00 (0.42–2.38)	
Episodes per Year				
≤0.35	50	16.0%	1.00	.010^a^
0.36–0.56	46	17.4%	1.48 (1.10–2.00)	
0.57–0.83	46	39.1%	2.20 (1.21–3.98)	
>0.83	50	34.0%	3.26 (1.34–7.95)	
Total Number of Depressive Episodes per Year				
≤0.15	47	10.6%	1.00	.002^a^
0.16–0.28	49	24.5%	1.63 (1.19–2.22)	
0.29–0.52	49	32.7%	2.65 (1.43–4.94)	
>0.52	47	38.3%	4.33 (1.70–11.00)	
Patients Without Lithium Treatment				
Total Number of Episodes				
≤5	58	24.1%	1.00	.090
6–8	42	16.7%	1.28 (0.96–1.70)	
9–12	47	27.7%	1.63 (0.92–2.89)	
>12	45	37.8%	2.09 (0.89–4.91)	
Years Without Lithium Treatment				
≤4.25	48	20.8%	1.00	.401
4.26–9.5	49	28.6%	1.13 (0.85–1.51)	
9.6–18.25	47	27.7%	1.28 (0.72–2.28)	
>18.25	48	29.2%	1.45 (0.61–3.45)	
Episodes per Year				
≤0.57	47	21.3%	1.00	.616
0.58–1	61	27.9%	1.08 (0.81–1.44)	
1.01–1.58	36	33.3%	1.16 (0.65–2.07)	
>1.58	48	25.0%	1.25 (0.52–2.97)	
Patients on Lithium Treatment				
Total Number of Episodes				
0–1	64	17.2%	1.00	.002^a^
2–3	40	22.5%	1.59 (1.18–2.13)	
4–7	46	23.9%	2.52 (1.40–4.52)	
>7	42	47.6%	4.00 (1.66–9.62)	
Years on Lithium Treatment				
0–5	53	28.3%	1.00	.789
6–11	45	24.4%	0.96 (0.72–1.28)	
12–20	49	28.6%	0.93 (0.52–1.64)	
>20	45	24.4%	0.89 (0.38–2.10)	
Episodes per Year				
≤0.11	48	12.5%	1.00	.002^a^
0.12–0.32	47	25.5%	1.66 (1.22–2.27)	
0.33–0.6	50	24.0%	2.77 (1.48–5.17)	
>0.6	47	44.7%	4.61 (1.81–11.76)	
S-Lithium With Episodes, mmol/L				
≤0.52	39	25.6%	1.00	.282
0.53–0.59	37	24.3%	1.19 (0.87–1.63)	
0.6–0.7	41	31.7%	1.41 (0.75–2.67)	
>0.7	37	35.1%	1.68 (0.65–4.36)	
S-Lithium Without Episodes, mmol/L				
≤0.54	46	17.4%	1.00	.393
0.55–0.62	51	33.3%	1.14 (0.85–1.53)	
0.63–0.68	49	26.5%	1.29 (0.72–2.33)	
>0.68	46	28.3%	1.47 (0.61–3.55)	

AOO, age of onset; OR, odds ratio; S-lithium, serum lithium; SA, suicide attempt; ULR, univariate logistic regression.

a Statistical significance level.

Table 5 shows MLR of variables with *p* < .1 in ULR. Significant risk factors revealed by this analysis were AOO (*p* = .001) and total number of depressive episodes per year (*p* = .003).

**Table 5:** MLR Analysis of Risk Factors (*p* < .1) for SAs in Bipolar Disorder

	*n*	SA, %	MLR	*p*
			OR (95% CI)	
Gender				
Female	112	32.1%	1	.078
Male	65	23.1%	1.96 (0.93–4.15)	
Age Category, Years				
≤46	47	38.3%	1	.622
47–58	46	26.1%	0.92 (0.65–1.29)	
59–68	48	29.2%	0.84 (0.42–1.67)	
>68	51	13.7%	0.77 (0.28–2.16)	
AOO, Years				
≤21	50	44.0%	1	.001^a^
22–30	53	30.2%	0.50 (0.34–0.76)	
31–38	48	18.8%	0.25 (0.11–0.57)	
>38	41	9.8%	0.13 (0.04–0.43)	
Total Number of Depressive Episodes per Year				
≤0.15	47	10.6%	1	.003^a^
0.16–0.28	49	24.5%	1.71 (1.21–2.41)	
0.29–0.52	49	32.7%	2.91 (1.46–5.81)	
>0.52	47	38.3%	4.96 (1.76–14.00)	

AOO, age of onset; MLR, multivariate logistic regression; OR, odds ratio; SA, suicide attempt.

a Statistical significance level.

## Discussion

Similar to previous studies, we found that 26.6% of 192 BPs performed an SA during a period of approximately 25 years (3, 4, 5, 6). We also found, consistent with past research, that SA was more common in females and in younger age groups with earlier AOO in BD (9,12, 13, 14, 15, 16,20, 21, 22, 23, 24, 25, 26, 27, 28, 29).

Noncompliant behavior is a serious and common problem while on lithium treatment in BD. An earlier study showed that poor compliance in BPs varied between 12% and 64% (64). In another study, up to 85% were reported to have a poor compliance in a patient group treated with lithium (65). Overall, in our study of BPs, we found that almost 1 of 2 demonstrated poor compliance. However, it should be pointed out that BPs with SAs had significantly poorer compliance compared with those without SAs. Noncompliance is a major barrier to effective lithium treatment. Further research is required, but more regular S-levels and earlier intervention to promote adherence may improve lithium efficacy.

Several studies (18, 19, 20,35,66) have found that BPs with an FH of AD have a more severe form of the disorder (earlier AOO, increased number of episodes of the disease, more hospitalizations, and SAs). In our study, an FH of first- and/or second-degree relatives of AD was found to be more common among BPs with SAs. An FH of a first-degree relative of AD was also more common in BPs with SAs compared with patients without SAs. Our results suggesting that an FH of AD is a risk factor for SA are consistent with previous studies (10,13,30,32, 33, 34). We also found that an FH of suicide was more common in BPs with SAs compared with patients without SAs, which is also consistent with a previous study (30). The long-term follow-up time of our study adds weight to the importance of the association between FH of AD and BD when assessing patients with depression.

We found that BPs with SAs had more total episodes and episodes per year compared with BPs without SAs. This could be explained by more depressive episodes and depressive episodes per year in BPs with SAs. It should be noted that there was no difference in follow-up time between BPs with or without SAs. BPs with and without SAs were observed during a mean period of 12 to 13 years before lithium treatment started. The only difference between the 2 groups was that BPs with SAs had more depressive episodes during the period before lithium treatment compared with those without SAs.

Both groups were treated with lithium for approximately 12 years. On lithium treatment, BPs with SAs had a higher number of episodes of the disease and a higher frequency of episodes per year compared with BPs without SAs. BPs with SAs also had a higher number of depressive episodes, depressive episodes per year, hypomanic episodes, and hypomanic episodes per year. The difference between the groups could mainly be explained by a high number of depressive episodes and a high frequency of depressive episodes per year among BPs with SAs. Because BPs with SAs are likely to have a more severe form of BD, it is important that the latency time from onset to when lithium treatment is instituted is kept as short as possible. Since BPs with SAs have more depressive episodes and a high frequency of depressive episodes per year compared with BPs without SAs, the BPs with SAs is possibly considered to suffer from a unipolar depression rather than a BD and therefore the initiation of a lithium therapy may be unnecessarily delayed in these cases.

In our study, we found that lithium treatment was associated with a reduction in episodes of the disease, episodes per year, depressive episodes, and depressive episodes per year both among BPs with and without SAs. Therefore, it should be noted that in our sample of BPs with SAs, the vast majority (3 of 4) had good protection against SAs by the support of lithium treatment. However, BPs with SAs showed a poorer treatment response to lithium therapy and, especially, a higher frequency of nonresponders. S-lithium levels were similar in the groups and therefore could not explain the observed differences between the 2 groups of BPs. Therefore, this observation suggests that as in other investigations, BPs with SAs might have benefitted in general from striving to achieve higher S-levels of lithium (67, 68, 69). However, this possibility has to consider that drug tolerance might then be hampered by increasing the problem with adverse drug reactions.

The observed differences in lithium response between BPs with and without SAs cannot be explained by time until initiation of lithium treatment or to define the BD diagnose. Both groups were generally treated late in the disease with lithium (approximately 11 years from onset) and fulfilled the criteria for BD late after onset (6–8 years) during the establishment of the disorder. It has also been pointed out previously that patients with major ADs often receive the appropriate treatment and diagnosis very late in the course of the disorder [for review, see (70)].

In a previous study of BPs while off and on lithium treatment, the authors estimated that approximately 90% of SAs occurred during periods with no lithium therapy (71). In our group of 51 BPs with SAs, almost 3 of 4 performed SAs before initiation of lithium treatment. Of those patients, none performed a second SA after introduction of lithium treatment. As previously mentioned, some of the SAs in these situations could perhaps have been prevented if patients had received lithium treatment in the earlier stage of the disorder. Lithium was not effective in preventing SAs in 25% of those treated. In our sample, this group showed higher S-levels of lithium (0.67 vs. 0.61 mmol/L) indicating adequate treatment. Although not evidenced by the current data, it is possible that the group who performed SA while on lithium therapy represents an even more severe form of BD with a less protective effect of lithium drug therapy as prophylaxis. It has been estimated that lithium treatment may be ineffective in as many as one-third of cases, depending on how diagnosis and effectiveness of treatment are defined. This underlines the fact that further research is required to better personalize treatment.

In short, the ULR and MLR analyses in our study show that AOO and a high total number of depressive episodes per year are the most important risk factors for SAs. These findings emphasize the importance in clinical practice of examining the possibility of presenting a diagnosis of BD early on. Consistent with this, it is also strongly motivated to be followed up with an early introduction to lithium prophylaxis to prevent suicidal behavior when AOO and a high total number of depressive episodes per year are present.

Observational studies such as the current one have the inherent problem that all findings can only be regarded as associative and not of causal origin. Nevertheless, the main advantage of the current investigation is the very long follow-up period of approximately 25 years. Here, long-term follow-up, both pre- and postlithium commencement, standardized assessment processes, and restriction of the sample to BPs only, are all to be considered separate strengths in view of a scientific research approach. On the other hand, in a broader perspective of where these patients may present in an everyday clinical setting, the representation of details in our findings are possibly not as obvious as in the pure scientific context. Dropout in longitudinal studies is a common problem, but this is not necessarily a limitation of our study, because the number of BPs (*n* = 192) and the follow-up period of 25 years is, to the best of our knowledge, one of the largest populations being studied in this way.

The possibility of the occurrence of multiple significance errors was considered, because this may be another limitation of the current study. However, the differences between the 2 groups in this follow-up study are purely descriptive and not inferential, and therefore corrections for multiple comparisons have not been made. Nevertheless, many of the significant variables are correlated. Moreover, because the parameters obtained in most cases were regarded as very different and most likely unrelated, we had yet another reason not to correct the statistical analysis for the risk of multiple erroneous significant findings. Such interventions, for example using the Bonferroni correction algorithm, may instead risk underestimating existing significant differences, thereby introducing a new bias.

### Conclusions

BPs with SAs have a more severe form of BD and can be characterized by the fact that they generally present at a younger age, are more frequently female, have an earlier AOO, more often have an FH of AD and suicide, have a higher number of episodes of the disease and a higher frequency of episodes per year, and have a higher number of depressive episodes and a higher frequency of depressive episodes per year. BPs with AD also have a less protective effect of lithium treatment, with a higher number of depressive episodes, hypomanic episodes, and a higher frequency of depressive and hypomanic episodes per year while on lithium treatment. Overall, our results emphasize the need for and the importance of long-term follow-up studies of BPs and SAs in the future, if possible, with a prospective design.

## Supplementary Material

Key Resources Table
